# An analysis of extensible modelling for functional genomics data

**DOI:** 10.1186/1471-2105-6-235

**Published:** 2005-09-27

**Authors:** Andrew R Jones, Norman W Paton

**Affiliations:** 1 School of Computer Science, University of Manchester, Manchester, UK

## Abstract

**Background:**

Several data formats have been developed for large scale biological experiments, using a variety of methodologies. Most data formats contain a mechanism for allowing extensions to encode unanticipated data types. Extensions to data formats are important because the experimental methodologies tend to be fairly diverse and rapidly evolving, which hinders the creation of formats that will be stable over time.

**Results:**

In this paper we review the data formats that exist in functional genomics, some of which have become *de facto *or *de jure *standards, with a particular focus on how each domain has been modelled, and how each format allows extensions. We describe the tasks that are frequently performed over data formats and analyse how well each task is supported by a particular modelling structure.

**Conclusion:**

From our analysis, we make recommendations as to the types of modelling structure that are most suitable for particular types of experimental annotation. There are several standards currently under development that we believe could benefit from systematically following a set of guidelines.

## Background

The advent of large scale approaches investigating biological systems has generated a requirement for standard data formats that has been recognised by the bioinformatics community for several years. It is a major challenge to create standards that are stable and "future proof" for considerable lengths of time. In this document, we review the models associated with standard data formats for microarrays, proteomics and metabolomics (collectively known as functional genomics). The experimental techniques in these areas are evolving rapidly, different laboratories use different instruments and software, and a single experiment can produce a wide range of heterogeneous data types. This causes problems because data produced in one laboratory often cannot be interpreted by other groups or compared with other data sets produced in a different setting. Proposals have been made for data standards for microarrays (MAGE-ML [1]), protein-protein interactions (the Molecular Interaction format [2]), mass spectrometry (most recently mzData [3] and mzXML [4]), and protein separation proteomics (PEDRo [5]). There have also been proposed extensions to MAGE-ML to accommodate other types of experiment (FGE-OM [6] and SysBio-OM [7]). Data standards for metabolomics are at an early stage, but there are three models that could contribute to a data standard: SysBio-OM models metabolome data arising from NMR (nuclear magnetic resonance) and mass spectrometry; CCPN [8] is a comprehensive model of NMR data for macromolecules; and ArMet [9] covers data arising from metabolomic studies on plants.

Each proposal has been developed using various modelling strategies that enable unanticipated types of data to be encoded or to allow the model to be extended in the future. This *extensibility *of the models is an essential component because new functional genomics techniques are frequently developed, new instruments and software have parameters and data types that must be stored, and there are no limits on the types of biological samples that can be tested. Sufficient annotation must be captured to allow data sets to be interpreted, queried and analysed, the context must be unambiguous, and the format should capture sufficient detail about the provenance of data.

In this paper, we first describe the modelling structures that allow for extensions and the tasks that may be carried out over biological data. We analyse how well each task can be supported if information is captured within one of the extensible structures. The following section examines the extensible structures employed in the current models and highlights potential problems, in terms of tasks that may not be adequately supported. We then make recommendations as to the modelling structures that best support the most important tasks for common parts of a functional genomics workflow, and discuss the relevance of such structures to the development of new data standards.

## Modelling constructs for extensibility

There are several structures that can be incorporated into models that allow additional types of data to be encoded without affecting the core schema. In this section, we first describe the modelling constructs that allow for extensibility, and then describe the kinds of tasks that may be performed over experimental data and its associated annotations, with a view to clarifying which extensibility features support which tasks.

### External ontologies

External ontologies are widely used for making extensible models. Ontologies are structured controlled vocabularies containing defined terms. Each term may be associated with a set of rules or relationships to other terms that allow logical questions to be asked of the ontology. Terms from an ontology can be imported into a model, which is advantageous because the term has a meaning beyond the scope of the source system. Furthermore, where there is a standard ontology, data produced by different laboratories will use the same terms, promoting greater uniformity across different systems. The following example demonstrates the use of ontologies within MAGE-ML:

<BioSource identifier="BioSource:Drosophila:OregonR" name="Drosophila strain, Oregon R">

   <MaterialType>

      <OntologyEntry category="MGED:MaterialType" value="Organism"/>

   </MaterialType>

   <Characteristic_assnlist>

      <OntologyEntry category="NCBI:Taxonomy" value="Drosophila melanogaster"/>

      <OntologyEntry category="Flybase:Genotype" value="wild type"/>

      <OntologyEntry category="Flybase:Strain" value="Oregon R"/>

   </Characteristic_assnlist>

</BioSource>

This example demonstrates the specification of a source of material (flies) of a particular strain. The element <Characteristic_assnlist> contains a set of characteristics of the biological material using terms obtained from two different controlled vocabularies. The FlyBase ontology [10] has a definition of the "wild type" genotype and the "Oregon R" strain of flies. The NCBI taxonomy [11] is used to specify which species is being studied. The definitions can be retrieved if required to ensure that the species, strain and genotype are unambiguously described.

### Name-Value-Type triples

Many of the data standards listed in the introduction have name, value, type (NVT) triples that allow additional parameters or data types to be added by the user which do not exist in a publicly available controlled vocabulary or ontology. "Name" stores the item that must be captured, "value" is the data value and "type" is a qualifier or unit. The following example is taken from the mzXML format:

<nameValue name ='heatedCapillaryTemperature' value='203.4' type='Celsius'>

In this example, there is an additional property (heatedCapillaryTemperature) that must be encoded in the data format but was not incorporated in the core schema. The parameter has a parent element that corresponds to the mass spectrometry device, demonstrating that NVT is usually context-sensitive.

### External files

Additional information not covered in the data schema can be captured in separate files that are referenced from the source document. Many data formats are encoded with Extensible Markup Language (XML), which is a fairly verbose format. In some instances, information is captured in separate tab-delimited files, spreadsheets, word processing documents or image files. For example, both MAGE-OM and PEDRo specify that image data should be stored in a separate file and referenced by a URI (Uniform Resource Indicator).

### Inheritance

**Figure 1:**
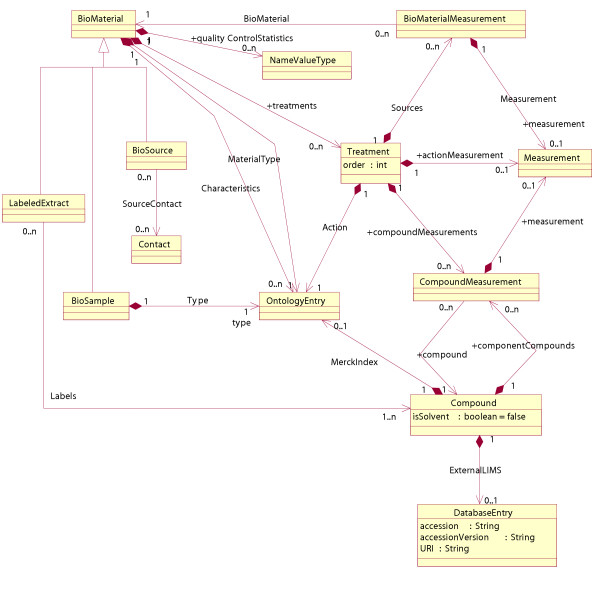
The BioMaterial package in MAGE-OM. There are three subclasses BioSample, LabeledExtract and BioSource of the superclass BioMaterial.

Inheritance is used in software engineering to reduce the size of a model and make explicit areas of overlap by re-using certain components. Models are often represented in the Unified Modeling Language [12] (UML), which facilitates the design of software systems in a platform independent manner. The example in Figure 1 demonstrates how inheritance has been used in MAGE-OM, the object model that is part of the microarray data standard. The classes LabeledExtract, BioSource and BioSample are all subclasses of the general class BioMaterial. The associations between BioMaterial and other classes are inherited by LabeledExtract, BioSource and BioSample. These three classes have additional properties that make them more specific than BioMaterial.

Inheritance could be used to make a model extensible by designing a set of generic classes that describe components shared across all possible domains that use the format. Such a data model could grow over time by the addition of new subclasses containing attributes that are specific to a particular domain or to a newly emerging technology. This would have the effect that previous versions of the standard should still be supported by software, and that the standard can continuously evolve. An example of a standard developed in this way is the Geography Markup Language [13] (GML), which contains a modular structure allowing developers to use the subsets of the model that apply to their domain of interest. Inheritance has also been used extensively in MAGE-OM, the microarray object model, and the model has been extended by the addition of new subclasses in FGE-OM and SysBio-OM that do not affect classes defined in MAGE-OM. While there is no current proposal in functional genomics for the development of an evolving standard, the release of the FGE-OM and SysBio-OM models raises interesting questions as to whether this may be a feasible methodology for defining an extensible data standard. In the results section, we define this kind of extensibility as *Extend Model Inheritance *(EMI).

## Tasks

We have identified a set of tasks that a data model must support for users. In some instances it is assumed that the data model has been implemented in a system, such as a database. The tasks are as follows:

• **Search**: performing of simple searches over the attributes coded in extensible structures to retrieve particular data sets.

• **Share**: sharing of data sets between different research groups.

• **Read**: manual reading of data files (or the extracted text) to understand the intention and execution of an experiment.

• **Repeat experiment**: the provision of sufficient detail on methods and protocols to allow an experiment to be repeated.

• **Compare experiments manually**: manually determine how similar different experiments are.

• **Compare experiments automatically**: automatically determine using a software system if two experiments are sufficiently similar to allow results to be directly compared. Although there are many other issues that may prevent automatic comparison of results, such as the use of incompatible accession numbers from different databases to identify the same objects.

• **Query**: querying the parts of an experiment that have been encoded in extensible structures to retrieve particular subsets of data or to ask more complex questions about the structure of the data.

• **Analyse**: performing of statistical or analytical processes over the data set.

• **Browse**: manually browsing the contents of a set of data files to find relevant experiments.

• **Populate**: creating data sets conforming to the standard.

There are also several tasks that fall into a different category, relating to the development and management of the model.

• **Modelling**: the ease with which the model can be created.

• **Data capture interface**: the cost to develop the user interface for populating the data format.

• **Query or browse interface**: the cost to develop the user interface for browsing or querying the data format.

• **Data management**: the cost of data management in terms of time for developers to implement changes to the database schema or additional software required for parsing.

• **Quality assurance**: the ability of the representation to prevent inclusion of an incorrect or imprecise value.

## Support for tasks

This section presents an analysis of how well each task is supported by the different extensible structures. The support for each task by each extension is described in Table 1 and Table 2. For many of the tasks, we differentiate between performing within an organisation (the *local case*) and the task being performed by a user from a different organisation from where the data are produced (the *non-local *case).

**Table 1:** The support for different tasks offered by different modelling structures: NVT (Name-Value-Type), ontologies, external files and extend model inheritance (EMI).

**Extension**	**Support for task: Search**
NVT	Different sources will differ in attribute and value, therefore good for local data because NVT can be used to encode arbitrary properties as long as local users are aware of the data types that can be searched. Poor for non-local searches, as inconsistent attributes and values are likely to be used.
Ontology	Okay if searched with exact matching terms; more difficult to support non-exact match because the search engine is unlikely to search within the ontology structure.
External file	Not good; there may be no access to the structure of the file. Only information retrieval style requests can be made.
EMI	Extensions can be searched locally but non-local searches will not be possible unless the extended models are shared.

**Extension**	**Support for task: Share**

NVT	Good for local sharing, poor for sharing externally because properties may be encoded in NVT in inconsistent ways.
Ontology	Good if terms agree (if the same ontology has been used).
External file	Okay if file is in a standard format, otherwise bad (information may be difficult to access).
EMI	Good for local sharing; cannot be shared externally unless the extended models are shared.

**Extension**	**Support for task: Read**

NVT	Generally good because writer can be expressive (NVT is better than plain text); only problem is misinterpretation if NVT is used inconsistently.
Ontology	Good because terms are well defined.
External file	Good if file is in a standard format, otherwise bad. Other software may be required to access the file, such as for images, archive files, spreadsheets and so on.
EMI	Good because writer can be as expressive as required.

**Extension**	**Support for task: Repeat Experiment**

NVT	Okay for local case (especially good if data capture is automated); in general it is a hard problem for the non-local case.
Ontology	Good for the non-local case. May be less good for local case if local terms are converted to ontology terms and cannot be converted back (ontology may not be able to express all local data in a lossless manner).
External file	Okay if file is in a standard format, otherwise bad.
EMI	Good for local case, poor for non-local case unless extensions are widely shared.

**Extension**	**Support for task: Compare experiments manually**

NVT	Okay, but inconsistencies could be problematic if data types are encoded differently in different settings.
Ontology	Good because terms are well defined and standard.
External file	Okay if the file is in a standard format that can be easily processed.
EMI	Generally good because the model developer can be expressive.

**Extension**	**Support for task: Compare experiments automatically**

NVT	Good for local case; not good for the non-local case because NVT is likely to have been implemented differently.
Ontology	Good (consistent representation from different experiments).
External file	Okay if data are stored in a spreadsheet or tab-delimited text and descriptive metadata are stored correctly within the data format, or if the external file is in a standard format that can be easily processed.
EMI	Good for local case; cannot be done for the non-local case unless the extensions are widely shared.

**Extension**	**Support for task: Query**

NVT	Worse than problem for search because queries are generally more precise.
Ontology	Generally good, but must query more than one language and the software for query evaluation may not be able to call out to a reasoning service (to make use of the ontology structure).
External file	Not good (it must be assumed that there is no access to structure).
EMI	Good for local case, cannot be queried non-locally unless the extensions are shared.

**Extension**	**Support for task: Analyse**

NVT	Not possible; generic analyses must not depend on such data.
Ontology	May not be relevant; analysis is not usually over ontology terms (but much better than NVT if it is).
External file	Okay if data are stored in a spreadsheet or tab-delimited text and metadata are stored correctly within the data format, or if the external file is in a standard format that can be easily processed.
EMI	Okay for local analysis but additional wrappers may be required to allow generic analysis software to access the data. Poor for non-local case as the format will have to be interpreted and software must be written.

**Extension**	**Support for task: Browse**

NVT	Okay (probably better than plain text).
Ontology	Good, less chance of misinterpretation than NVT.
External file	Not good unless file is immediately readable.
EMI	Good because writer can be expressive.

**Extension**	**Support for task: Populate**

NVT	Easy to populate but hard to enforce consistency.
Ontology	Easy as long as ontology is in place and easily accessible.
External file	Easy to populate but hard to enforce consistency.
EMI	Easy.

**Table 2:** The relationship between the development of systems to support a data standard and the different modelling structures that could be used: NVT (Name-value-type), ontologies, external files and extend model inheritance (EMI).

**Extension**	**Support for task: Modelling**
NVT	Near zero cost.
Ontology	Expensive (hard to develop ontology).
External file	No cost.
EMI	Fairly high cost because additional modelling in advance and the developer must understand the core model and how it can be extended.

**Extension**	**Support for task: Interface (for populating)**

NVT	Fairly easy as the code need not reflect the attributes, but difficult to ensure consistency as there is no explicit prompting from a controlled vocabulary.
Ontology	Some additional costs (importing ontology or calling an ontology service)
External file	Very easy (just upload the file).
EMI	Changes required to the interface to reflect the extensions unless the interface is created automatically from the model.

**Extension**	**Support for task: Interface (for query/browse)**

NVT	Few additional costs as the interface code need not reflect the attributes.
Ontology	Low cost as the queries can be generated from the model and ontology.
External file	The default is no functionality over the file otherwise extra coding is required which may be relatively costly.
EMI	Additional costs as interface code must be written to cover the extension unless the interface is generated from the model.

**Extension**	**Support for task: Data management**

NVT	Low cost as no changes are required to the schema.
Ontology	No changes to database schema but small additional costs because the ontology has to be stored locally or linked externally.
External file	No changes required to the schema but small additional cost because more than one storage mechanism must be managed (database and file system).
EMI	Changes are required to the schema, which are likely to be expensive.

**Extension**	**Support for task: Quality assurance**

NVT	None; terms should be used with caution. NVT cannot restrict the cardinality or possible values.
Ontology	Good because a domain value can be enforced.
External file	No constraint or value checking.
EMI	Some quality enforcement because there will be guidelines as to the types of extensions allowed to a model and the model will enforce constraints on the value stored.

## Extensibility in biological models

### MAGE-OM

A standard has been developed for microarray data, of which one part is an object model, called MAGE-OM (MicroArray and Gene Expression – Object Model), which is expressed in UML. The developers of MAGE-OM recognised that microarray technology was still evolving, that the types of experiments were fairly diverse, and that the biological samples on which experiments could be performed are practically infinite, yet all the information should be captured in a structure that would support many of the tasks described above. Therefore, several modelling constructs have been used in MAGE-OM to create a highly extensible object model.

### Ontologies in MAGE-OM

MAGE-OM has many specified places in which parts of external ontologies can be imported. Examples include the characteristics of biological samples, types of biological material or compounds, and taxonomic classifications of organisms. A term can be obtained from any ontology as long as the source of the term is specified. This allows the object model to be stable but the external ontologies can grow over time with contributions from domain experts to increase the coverage of the data standard. Changes to the ontology are unlikely to cause software to fail whereas most software is dependent on the structure of the object model.

### NVT triples in MAGE

The Extendable class in MAGE-OM has a relationship to a class (NameValueType) that has the attributes name, value and type (NVT). All other classes in MAGE-OM are subclasses of Extendable and inherit this relationship, allowing additional properties to be captured in NVT triples with no restrictions. In the various data repositories that support MAGE, there have been few, if any, reported uses of general NVT triples because there are usually specific classes that have been used to capture a particular concept. The inclusion of the NVT triple class could cause problems as experimental parameters encoded in this way could not be automatically compared with other experiments that have modelled parameters correctly and the values may not be capable of being queried.

### External files

MAGE-OM represents processed data, resulting from image analysis, in external files containing tab-delimited data. The model captures metadata to describe what each column refers to, which is essential to ensure that when the data files are re-analysed there should be no misinterpretation of what is contained within external files. This design is advantageous because tab-delimited data files are more compact than XML. MAGE-OM also allows external image files to be specified (the raw data from the experiment), as image files tend to be in standard formats that can be interpreted by widely available software.

### Extension to MAGE-OM through inheritance

There has been no formal attempt to evolve the MAGE-OM standard by the addition of new classes that inherit from parts of the core model, but there have been two proposals that have extended MAGE into other areas of functional genomics, called FGE-OM and SysBio-OM. Both models cover microarrays and proteomics, and SysBio-OM additionally covers metabolomics. In several places the two models have extended MAGE-OM through the use of inheritance. For example, both proposals include new subclasses of BioMaterial (shown in Figure 1) to model substances specific to proteome studies, such as spots on a two-dimensional gel and fractions from a column separation. The two models also create new subclasses of classes modelling a generic laboratory treatment, the inputs to the treatment and the output. A similar design is used in PEDRo (see below). It is interesting to note that several different designs have arrived at a similar method for specifying laboratory treatments, raising the possibility that MAGE-OM could become a standard that grows over time through the addition of new subclasses modelling inputs, treatments and outputs.

### PEDRo

#### Overview

The PEDRo (Proteomics Experiment Data Repository) model was released in early 2003 to stimulate community involvement in the development of a data standard for proteomics. PEDRo consists of an object model expressed in UML, which covers protein separation techniques, such as gel electrophoresis and liquid chromatography, and protein identification using mass spectrometry. Around the same time the Proteomics Standards Initiative (PSI [14]) was founded by the Human Proteome Organisation (HUPO) to develop data standards for proteomics in the context of protein-protein interactions and mass spectrometry (MS). PEDRo has been accepted as the working model of PSI for protein separation based experiments. PEDRo is divided into four sections capturing (i) the design of the experiment and source of material, (ii) protein separation, (iii) the experimental setup for MS, and (iv) database identification of proteins with MS data. The design methodology of PEDRo is significantly different from MAGE-OM. PEDRo has detailed classes containing attributes that specify exactly what data type should be stored in which position. The model is very tightly specified and it is unlikely that experimental annotation encoded in PEDRo would be open to widespread misinterpretation. However, the model is relatively rigid and cannot easily be extended to cover unanticipated data types. PEDRo does not utilise extensible structures to describe biological samples, and therefore cannot store a structured description of all types of sample that may be used in proteomics.

#### Ontologies, NVT and inheritance in PEDRo

PEDRo uses ontologies in a small number of positions, such as additional parameters for database searches or unanticipated types of laboratory treatment. There are no instances of ontology usage in the database implementation (PEDRoDB [15]), due to the lack of controlled vocabularies in the proteomics field at present. There are no positions at which NVT triples are employed in PEDRo. PEDRo uses inheritance by including superclasses that capture (i) the concept of a substance (Analyte) used in a proteomics experiment and (ii) the type of processing or technique used (AnalyteProcessingStep). An AnalyteProcessingStep takes instances of Analyte as input and output. The specific details of each processing step or substance are captured in subclasses. This design could in theory be extended by adding new subclasses of AnalyteProcessingStep and Analyte. An evolving standard may be possible, although the overhead of vetting, discussing and finalising additions to the model may be prohibitively costly.

#### External files

Images of electrophoresis gels are represented in separate files in PEDRo, which is an acceptable solution because most users will have software that can view the majority of image file formats. PEDRo also specifies that a file containing the input parameters for MS instruments or database searches can be specified. This could cause problems if the file is not in a standard format because it will support very few of the important tasks for the user, such as query or compare experiments automatically. If the file is a proprietary format, the information may not be readable or accessible to some users at all.

### Models for mass spectrometry

There have been several proposals in the past covering general MS data formats, including SpectroML [16] and ANDI [17]. We focus on three recent proposals for MS data standards: mzXML produced by the Institute for Systems Biology, mzData developed by PSI and AniML developed by ASTM [18] (an internationally recognised standards organisation). The mzXML format is a superset of the data formats produced by different instrument manufacturers, and software has been developed to convert many of the vendor specific data formats to mzXML. It is planned for future versions that controlled vocabularies will be used for vendor specific details, such as the name and type of instrument used. However, the current version of the mzXML schema does not use ontologies to capture additional information; instead, the options for terms are included within the schema. This design means that the schema can be used immediately with no additional resources required but that it cannot be extended to cover new types of technology without releasing a new schema. Additional information can be captured in the format using an NVT element that has no restrictions.

The mzData format has a similar goal to mzXML, namely to provide a single encoding of information from the different output formats produced by MS instruments. Controlled vocabularies will be used to populate many parts of the format including lists of instrument parameters, the detection mechanism, and the type of MS analysis. Supplementary information can also be captured for several objects in an element that captures the name of the object, the value and the simple data type (String, Boolean, float etc). This might cause problems because, as stated above, NVT triples may not be open to automated analysis. The source file from which the mzData file is created can be referenced using a URI. Source files are usually proprietary formats that cannot be processed by other groups. As such, there will be limited benefit in relating the mzData file back to its source, except for the purposes of local laboratory management.

The mzXML and mzData formats have very limited descriptions of the biological samples used in the experiment because it is intended that they will be used in conjunction with another data standard, such as the PSI-OM [19] model of proteome data. Various instrument parameters can be captured in both models using NVT triples, which could cause problems for querying or comparison of different data files. However, instrument parameters are unlikely to be used for searching or querying and rarely for analysis; therefore, it is possible that NVT triples are an adequate structure for encoding such information.

AniML is a model for analytical chemistry data, including the output from mass spectrometry, NMR and chromatography. AniML consists of a flexible core defined by an XML schema. There are extensions for different experimental techniques, which are XML instance documents, *rather than XML schemas*, defining the allowable values. This approach could be viewed as a combination between using inheritance and ontologies because specific terms are defined that should be used in particular places in the format. In this context, this is an extension of the core schema by providing more strict requirements in the form of controlled vocabularies. However, the controlled vocabularies are effectively hard-coded in the extensions.

### Convergence of mass spectrometry formats

It is essential that the three formats converge to some extent to allow standardisation of mass spectrometry data files. One of the main differences is the method in which controlled vocabularies are referenced. The mzData format can include references to an external list of terms with accession numbers. In contrast, mzXML includes the terms hard-coded within the schema, although it is planned for mzXML version 2 that external CV terms will be used. AniML has specific terms in the technology specific extensions. The advantage of placing the terms outside of the schema, as in mzData and in the AniML technology instance documents, is that changes can be made to the list of terms without releasing a new schema. This has the disadvantage that additional software is required to verify that external terms have been used correctly. The mzXML format can be validated using only a standard XML Schema parser but if new terms are required, a new version of the schema must be released.

There has recently been an agreement that the same terms will ultimately be used by mzData, mzXML and AniML. Furthermore, future versions of mzXML will include references to external vocabularies, hence becoming closer to mzData in structure. It should be possible to write software that converts data between the different formats, although it is unlikely that all formats will have exactly the same coverage. It is hoped that the different organisations continue to collaborate to bring about the unification of the formats.

### Molecular interaction format

A standard data format for protein interaction experiments, such as Yeast Two-Hybrid [20], has been developed by PSI called the Molecular Interaction Format (MIF), which is defined by an XML Schema. The first release of the format (level 1) covers the data that is available in most of the publicly accessible databases. PSI has developed a controlled vocabulary of terms which are used at specific places in MIF. An example term is the name of the experimental method but the format does not have a detailed description of the experimental protocols or the biological samples used. Descriptions of experimental protocols will be required in future versions because the results of protein interaction experiments are highly dependent on the technique used [21]. The Gene Ontology [22] (GO) will be used for describing genes and proteins and the NCBI Taxonomy will be used to standardise the names of species. Extensible structures may be less important for MIF because its primary use is the transfer of data between pre-existing databases. As such, the format's requirements are known in advance to some extent. If extensions are required, they can be accommodated in the next release of the standard.

### Metabolomics

There are three data models that have relevance for the metabolomics community: SysBio-OM, ArMet and CCPN. SysBio-OM is an extension of MAGE-OM with the addition of new classes to model NMR data that may arise in a metabolome investigation. ArMet is a proposal from the plant metabolomics community to capture the large volumes of data that are being produced as a result of GC-MS (Gas Chromatography – Mass Spectrometry) experiments on plants. CCPN is a data model produced by the NMR community to capture details of the starting sample, the input parameters and the output from the instrument. CCPN could be used to capture metabolome data because NMR is a commonly used technique for analysing the metabolites present in a sample.

SysBio-OM has a close correspondence with MAGE-OM, and shares the same kinds of extensible modelling structures. Therefore, the comments about NVT, ontologies and external files for MAGE-OM are also relevant for SysBio-OM. CCPN contains a fairly detailed object model, and many classes have a large number of attributes that specify exactly the data types that can be captured. It is similar to the design of PEDRo in that it uses few extensible structures, although references to external databases for molecules or chemical compounds are allowed. Data files produced from CCPN are likely to be consistent and open to querying, although the format may need constant updates if there are changes in technology.

The ArMet proposal specifies that controlled vocabularies can be used for describing biological samples and chemical compounds but uses few of the extensible structures described above. The developers of ArMet suggest that the format may evolve and it could be extended through inheritance. If extensions are developed to the model, it is vital that they are widely publicised to prevent the development of different dialects of the format that cannot be compared.

## Results

**Figure 2:**
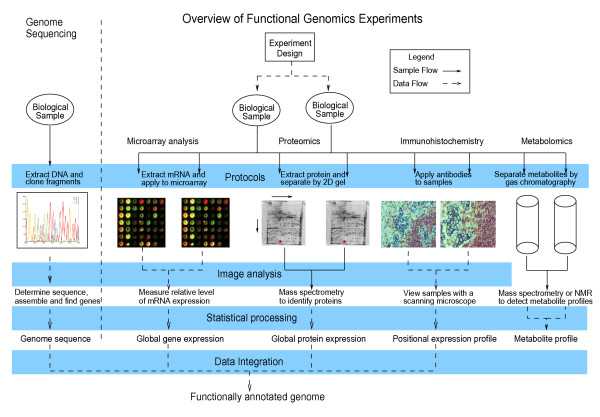
The shared components in different types of functional genomics experiments. The immunohistochemistry images were obtained from .

In this section we examine various parts of a generic functional genomics experiment (Figure 2 displays a summary of certain types of experiments), and determine the relative importance of each task listed above. We have identified the following areas that have highly similar annotation requirements across all types of experiment: the experimental hypothesis, the source of biological material, experimental protocols, numerical data and machine or software parameters.

### Experimental hypothesis

The purpose of a functional genomics experiment (the hypothesis) is typically to discover the genes, proteins or metabolites that are present or expressed in a sample of interest, or those that are altered in one set of conditions compared with another. The critical difference between the conditions must be open to searching and querying. The hypothesis is often the first text that will be viewed by someone accessing the data set to determine its relevance, and it is therefore of primary importance that it can be read and browsed.

The relative importance of each task is summarised in Table 3. We believe that querying, searching, browsing and sharing are of greatest importance for experimental hypotheses. If these components are to be captured in extensible structures, ontologies are the only option that allow all these tasks to be well supported. NVT triples should not be used because querying or searching this information would be hindered.

**Table 3:** Importance of tasks for annotation about an experimental hypothesis (*hypothesis unlikely to be analysed).

**Task**	Search	Share	Read	Repeat	Comp man	Comp auto	Query	Analyse	Browse	Populate
Importance	High	High	High	Med	High	Med	High	Low*	High	High

### Source of biological material

The source of material is a critical part of the experimental annotation because the results of a functional genomics investigation only have any validity within the context of the sample from which they were generated. It is important that biological samples can be queried or searched to enable users to retrieve relevant data sets, and samples must be described in a manner that allows automated comparison of experiments (Table 4). We stated that querying and searching are best supported over ontology terms and that NVT triples or external files will cause problems. It seems appropriate that efforts are focussed on designing ontologies that contain terms to describe samples, for instance as extensions to the MGED Ontology [23].

**Table 4:** Importance of tasks for descriptions of biological material (*analysis unlikely to be over biological samples).

**Task**	Search	Share	Read	Repeat	Comp man	Comp auto	Query	Analyse	Browse	Populate
Importance	High	High	High	Med	Med	High	High	Low*	High	High

### Experimental protocols

The basic protocols employed in an experiment have fairly similar semantics across all functional genomics experiments, and similar representations of experimental protocols are present in several models. It is unlikely that fine details of protocols will often be searched or queried but the protocol text must be easily readable to allow manual comparison of results (Table 5). A well structured description of protocols may allow results from different experiments to be compared automatically. If NVT is used to express protocols, reading and manual comparison of experiments will be fairly well supported but automatic comparison will not be possible. The use of ontologies to capture protocols would improve facilities for automated comparison of experimental results, but the cost to model all types of protocol with controlled terms may be prohibitively high. There would be limited benefit storing protocols in external files, such as a word processing documents, compared with storing plain text within the core data format (apart from formatting).

**Table 5:** Tasks for experimental protocols.

**Task**	Search	Share	Read	Repeat	Comp man	Comp auto	Query	Analyse	Browse	Populate
Importance	Low	Med	High	High	Med	High	Low	Low	Med	High

### Numerical data

The importance of the provision of support for tasks over numerical data is presented in Table 6. Examples in this context could be raw or processed data, such as the ratios of fluorescence from a microarray scan, or quantification data from a proteomics experiment. The important tasks over numerical data are analyse, share and query. There may be metadata that describes the semantics of the values, which should be described using ontology terms if possible to allow queries over the data. The actual values could be stored in an external file, such as tab-delimited text or a spreadsheet over which standard analyses can usually be performed.

**Table 6:** Tasks for numerical data.

**Task**	Search	Share	Read	Repeat	Comp man	Comp auto	Query	Analyse	Browse	Populate
Importance	Med	High	Low	Low	Low	High	Med	High	Low	High

### Machine parameters

Many types of instrument and software have a set of input parameters. The most important uses for the parameters are to allow the experiment to be repeated, and to underpin automated comparison of results between two or more experiments. In many cases the equivalence of results can only be established if all the parameters are equal. Tasks such as query, search, read or browse are much less relevant (Table 7). Non-local repetition of experiments requires encodings using ontologies, and NVT should only be used to allow local repetition. However, it is unlikely that controlled vocabularies, containing parameters from all types of instrument, will exist. In this case, the use of NVT is preferable to storage in external files or in extensions to the model, because NVT should allow experiments to be compared manually, and the parameters can be accessed more easily if encoded in NVT rather than in a proprietary format.

**Table 7:** Tasks for machine parameters.

**Task**	Search	Share	Read	Repeat	Comp man	Comp auto	Query	Analyse	Browse	Populate
Importance	Low	Med	Med	High	Low	High	Low	Low	Low	Med

## Discussion

It is important that data standards are created that allow flexibility in the data types that can be captured. This issue is particularly important for experiments such as proteomics, in which large volumes of data are created but the experimental methodology is frequently changing. Data models must allow for extensions that cover new technologies, otherwise a "data standard" will only cover a subset of experiment types that exist. New proposals for standards would continue to arise, or intrusive changes would be required on a regular basis. Developers should also be wary of creating models that are overly general or give users too many options for how information can be encoded. In these cases, dialects of models could arise where information can be encoded sufficiently but interpretation by different groups is difficult. We have examined data structures that allow for extensibility, identifying how well a set of tasks can be supported by data encoded in each type of structure (Table 1). The general findings are as follows. For most parts of experimental annotation, ontologies give significant advantages to users because the standardisation of terms allows for improved searching and querying, and reduces the chance of terms being misinterpreted. Ontologies also allow software to perform automated analysis to determine the similarity between different experiments. The disadvantage is that ontologies are expensive for developers to create, and present some additional costs to the user in data management (Table 2). Furthermore, ontologies will never be able to cover all the terms required by all users, because ontology development will always lag behind the creation of new experimental techniques, software or instruments. There are also issues of consistency and maintenance of ontologies which are unlikely to be resolved by official standards organisations due to the costs involved.

NVT triples give considerable flexibility to the user and they are preferable to the storage of parameters in proprietary formats because NVT encodings can be read and browsed. NVT triples are difficult to search or query though and they should not be used for data types that will be used frequently to retrieve data sets. It is important that data formats are well documented to ensure that there are guidelines for "reasonable" usage of NVT triples. In several of the data formats supplementary information about objects can be provided using ontology terms where they exist or NVT for user-defined terms. If the same user-defined terms are used frequently by different groups, this can be a mechanism for discovering new terms that should be updated in the ontology.

External files are an acceptable solution for images and for tab-delimited data if there are facilities within the core schema for capturing metadata describing the data type in each column in the file. External files should not contain information that is required for querying and searching, and they should be in a standard format that all users can process easily.

We have suggested that a data model could be developed incrementally using inheritance to add new classes that capture technology specific details. A model developed using this strategy must include generic classes that capture the concept of a laboratory technique, biological substances, raw and processed data. The core of the model should not contain details that are specific to a particular technology. Any extensions that are developed must be carefully managed to ensure that parallel development of different models covering a single domain is avoided. There must be strict guidelines for the kinds of extensions that are allowed and good documentation describing the intended usage of the core model.

We briefly touched on the issue of data quality. Data quality is a very broad concept that can be measured in a variety of ways relating to the consistency and credibility of a record [24]. Consistency can be classified into format and value consistency. Format consistency comprises rules for how the data should be parsed in terms of simple data type usage (string, integer or float), cardinality and so on. Format consistency could be verified over ontologies (if rules about the allowed syntax are encoded in the ontology) and extensions to the model, but only to a limited extent on NVT and not at all over external files. The use of an ontology is the only extensible solution which allows verification of whether values meet semantic rules (value consistency), for instance whether a genuine taxonomic name has been given for a "species" data type. Other aspects of data quality, such as credibility, often depend upon domain specific knowledge and they are difficult to control at the level of model development. The result is that sufficient annotation must be stored in structures that can be browsed, searched or queried to allow users to make judgements about data quality, and that data are open to statistical analyses.

## Conclusion

We have presented a classification of structures that allow for extensibility within models that are used to create standard file formats for functional genomics. We hope that the classification will help to guide the development of new data models and standards. The first version of a protein separation standard will be released by PSI within the next year, the second version of the microarray standard MAGE-OM is also under development, and metabolomics standards are being discussed. The guidelines we have presented should maximise the potential use of data sets, while allowing good expression of the data semantics as required by the users of each format.

## Methods

The modelling constructs were identified by reading the published literature and the technical documentation for each of the current proposals. We examined data repositories and software that have implemented a format to elucidate the tasks that are frequently performed. The results have been generated by cross-referencing the most important tasks for specified parts of an experiment with the modelling structures that can support those tasks.

## Authors' contributions

The analysis was performed jointly by AJ and NP. AJ drafted the manuscript and revisions were made by NP.
